# Experimental and Numerical Investigations in Shallow Cut Grinding by Workpiece Integrated Infrared Thermopile Array

**DOI:** 10.3390/s17102250

**Published:** 2017-09-30

**Authors:** Marcel Reimers, Walter Lang, Gerrit Dumstorff

**Affiliations:** Institute for Microsensors, -Actuators and -Systems (IMSAS), Microsystems Center Bremen (MCB), University of Bremen, 28359 Bremen, Germany; mreimers@mailhost.informatik.uni-bremen.de (M.R.); wlang@imsas.uni-bremen.de (W.L.)

**Keywords:** IR measurement, grinding characterization, sensor integration

## Abstract

The purpose of our study is to investigate the heat distribution and the occurring temperatures during grinding. Therefore, we did both experimental and numerical investigations. In the first part, we present the integration of an infrared thermopile array in a steel workpiece. Experiments are done by acquiring data from the thermopile array during grinding of a groove in a workpiece made of steel. In the second part, we present numerical investigations in the grinding process to further understand the thermal characteristic during grinding. Finally, we conclude our work. Increasing the feed speed leads to two things: higher heat flux densities in the workpiece and higher temperature gradients in the material.

## 1. Introduction

The machining of steel is still the basis for making construction elements. Grinding is one of the cutting processes usually used for precision machining and for a finishing process. Especially when it is used as a finishing process, the final properties are defined by this machining step. Giving a construction element a defined concentricity, flatness or a low surface roughness [1] is state of the art. However, since grinding can lead to high temperatures on the surface of the steel as well as the subsurface zone, these temperatures can be a critical thing; microcracks can occur [2], the microstructure can be transformed (e.g., austenite to martensite) or the hardness can be modified [3]. One of the most important things in present machining research is to understand the dissipation of energy during machining and how this energy changes the material properties in the subsurface zone (the zone from the surface up to two millimeters into the workpiece) [4,5]. To investigate this question, the flow of energy in the workpiece must be examined. So far, different approaches have been presented in the literature to measure temperatures during grinding. One approach is the integration of sensors in the workpiece such as thermocouples [6,7,8,9] or thin film resistive structures [6,10]. However due to the integration, the heat flux is influenced and the response time might be high, especially in the case of thermocouples in millimeter size. To measure the surface temperature of the workpiece during grinding, infrared cameras have been used [6,11,12,13,14]. The major disadvantage is that only dry grinding processes can be characterized because it is impossible to measure in under the presence of a liquid lubricant. However, since a lubricant is usually used in grinding, industry-oriented grinding processes cannot be investigated.

Our idea is the integration of an infrared thermocouple array in the workpiece to measure the temperature distribution during grinding with lubricant. The grinding process itself is setup in a way that thin layers in the range of 20–40 μm are removed. As this is usually a finish process and thermal damage in the subsurface zone of the constructive element should be avoided, the energy flowing in the workpiece, as well as the occurring temperatures, are of particular importance. We will show the capabilities of our measurement setup. Using finite element modeling, we calculate the heat distribution in the workpiece. Finally, the results of the numerical and experimental evaluation will be discussed.

## 2. Measurement Setup

**Figure 1:**
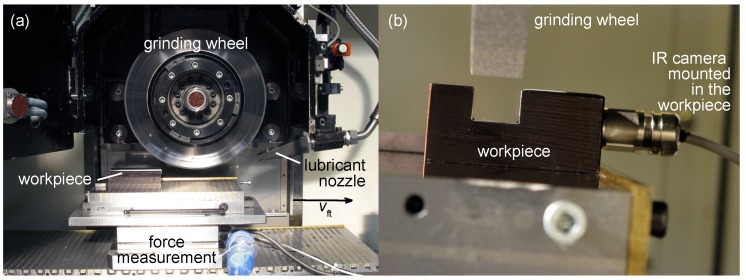
(**a**) Workpiece in the grinding machine; the table with the workpiece is moving to the right and the grinding wheel with the lubricant nozzle is fixed (**b**) Workpiece with integrated IR sensor mounted in the grinding machine (grinding direction = out of plane).

For our setup, the steel 42CrMo4 (AISI 4140) is used. To measure the temperature distribution during grinding, we used an infrared thermopile array (16 × 4 pixel IR array, HTPA 16 × 4R1, Heimann Sensor, Dresden, Germany). The thermopile array is mounted on a connector which is screwed in the prepared workpiece as seen in Figure 1. From the sensor, a cable leads to an interface receiving the thermopiles data via Inter-Integrated Circuit (I2C) bus. From the interface, data is send to an external computer via an User Datagram Protocol (UDP). The sensor is calibrated by the manufacturer. It has an internal microprocessor and a temperature sensor to ensure correct measurement.

Grinding was performed on an industrial surface grinder (Blohm Profimat 412 HSG 2005, Blohm, Hamburg, Germany). Characterization is done by grinding a groove with a width of 20 mm in the steel. White mineral oil is used as cooling lubricant. The grinding wheel is made of corundum.

**Figure 2:**
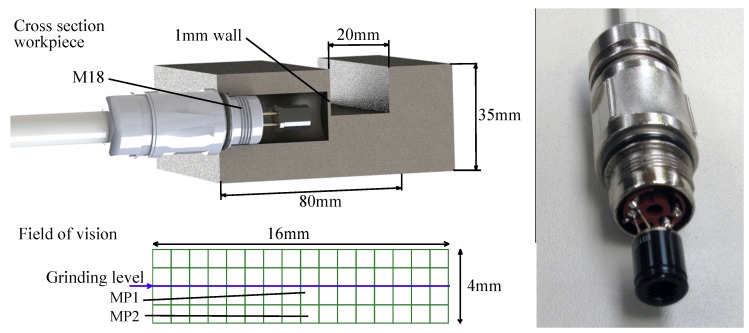
The **left** side shows a cross section of the workpiece with the camera and the field of vision of the sensor. On the **right** side, there is the IR camera with the M18 circular connector.

To produce a reliable distance between the grinding wheel and the sensor, a groove and the hole for the connector with the sensor was milled into the workpiece. An integrated seal from the connector prevents the sensor from grinding fluid. The sensor mounted in the workpiece is shown in Figure 2. For reliable measurements, the sensor must be placed in the same direction at every measurement. This was ensured with a mark on the connector. To ensure a reliable emissivity of the surface, it is coated with a black paint, having a defined emissivity of ε = 0.98. The temperature is then automatically corrected with the emissivity coefficient by the measurement software. The measured surface is placed 1 mm next to the side of the grinding surface as shown in Figure 2. The field of vision of the sensor was calculated to be 16 mm by 4 mm. Thus, one pixel is equal to 1 × 1 mm^2^ on the workpiece surface.

The field of vision and the grinding level are shown in Figure 2. The level of the grinding surface is right in the middle of the field of vision from the sensor. A wall with a thickness of 1 mm is necessary to protect the sensor from the grinding wheel. On the other hand, the sensor is close to the contact zone. However, due to the drilling hole for the camera, the heat distribution will be changed. This will be focused on Section 3. For our experiments and numerical investigations, we are focusing on the two measurement points shown in the field of vision in Figure 2. On one hand, we do not expect a significant influence of those points regarding boundary effects in comparison to the edges of the drilling hole. On the other hand, angle errors between the camera and grinding level will have a minimal influence since they are close to the rotation axis of the sensor.

Besides the temperature measurement, we also did a force measurement to get the tangential force $F_{t}$ during grinding. With the help of this, the total amount of energy occurring during grinding can be calculated. This will be explained in Section 3. To measure $F_{t}$, the workpiece is mounted on a force measuring platform.

In the grinding process, we vary two parameters. The first parameter is the infeed $a_{e}$ which defines the depth of cut. The second parameter is the specific removal rate $Q_{w}^{\prime }$. It defines the volume of the material, which will be removed in a specific time (independent of the grinding width). The specific removal rate is a function of the infeed $a_{e}$ and the tangential feed speed $v_{ft}$:

(1) $$Q_{w}^{\prime }=a_{e}v_{ft}$$

To change the specific removal rate $Q_{w}^{\prime }$ at a constant infeed $a_{e}$, the tangential feed speed $v_{ft}$ must be adapted. A higher $Q_{w}^{\prime }$ should increase the temperature in the workpiece. We want to investigate the occurring temperatures and heat dissipation at low infeed and low specific removal rate. As mentioned in the introduction, grinding is usually a finishing process, where thin layers of a few ten microns are removed to achieve precise geometry. Thus, we do a parameter study for $a_{e}$ = 20, 30 and 40 μm and $Q_{w}^{\prime }=$ 1.5, 3.0 and 4.5 $\frac{{\mathrm{mm}}^{3}}{\mathrm{mm}s}$, with the corresponding feed speeds using Equation (1).

**Figure 3:**
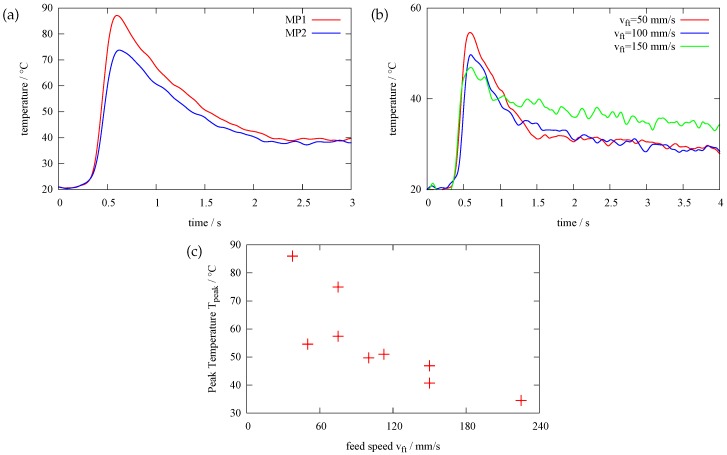
(**a**) temperature over time for $a_{e}=$ 40 μm and $Q_{w}^{\prime }=$ 1.5 $\frac{{\mathrm{mm}}^{3}}{\mathrm{mm}s}$ according to the measurement points MP1 and MP2 in Figure 2. (**b**) temperature over time at MP1 for $a_{e}=$ 20 μm different velocities of the workpiece (**c**) Measured peak temperature versus feed speed.

**Figure 4:**
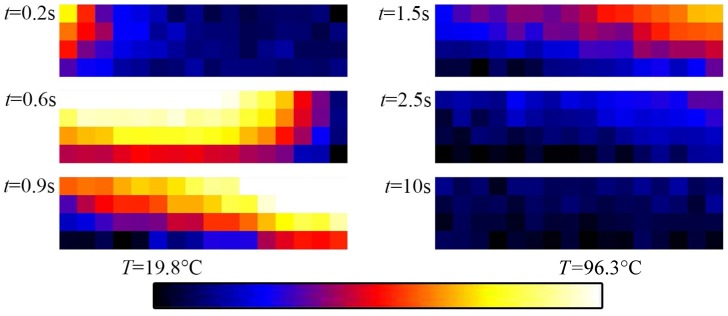
Photos taken by the IR camera at different times for $a_{e}=$ 40 μm and $Q_{w}^{\prime }=$ 1.5 $\frac{{\mathrm{mm}}^{3}}{\mathrm{mm}s}$ (see Figure 3a).

The temperature distribution at a specific time during different grinding stages is shown in Figure 3 and Figure 4. The graph in Figure 3a shows the distribution of two measurement points, namely MP1 and MP2 (see Figure 2), during one grinding stage. In the beginning, at t= 0.2 s there is a sharp rise in the temperature, reaching a peak of T = 88 °C at t = 0.6 s for MP1. The heat generated during grinding leads to sharp temperature rise in the workpiece. The temperature at MP2 is T = 72 °C and consequently lower because the measurement point is further away from the contact zone. After, the temperature has reached the peak it declines. On one hand, the steel acts as a heat sink and due to thermal conduction, the heat distributes in the steel. On the other hand, heat dissipates in the lubricant and the steel rapidly cools down. The shape of the curve during cool down (t> 0.6 s) looks like a typical cooling process. Figure 3b shows the measurement results for three different velocities of the workpiece. If we increase the velocity, the peak temperature at the point of measurement decreases. This is due to a shorter dwell time of the grinding wheel at one specific point on the grinding surface. In this case, the heat does not penetrate the workpiece very deep and the heat is immediately taken away by a fresh and cool lubricant. Regarding Figure 3c, there is a trend regarding all measurements: If we increase the feed speed of the workpiece, the peak temperature in the workpiece at the point of measurement decreases. To explain this unexpected result, we must look at the heat distribution during grinding with the help of a numerical model.

## 3. Numerical Modeling of Temperature Distribution

In this section, we model the sensor response by the help of finite element methods. With this, we want to get further information about the grinding process and to explain the occurring temperatures and gradients of different feed speeds.

In the contact zone, where the grinding wheel and the workpiece are in contact, heat is generated. This energy is dissipating in the chip, the grinding wheel, the lubricant and the workpiece. In our work, we focus the energy $q_{w}$ which is dissipating in the workpiece, leading to thermal treatment of the steel. The heat flux density out of the workpiece is mainly defined by the lubricant. At low removal rates, only a small amount of energy is dissipates in the chip and the grinding wheel. In our approach this three energies are summarized in one boundary condition, defined by a parameter study and a validation of the model. In general a convective heat transfer coefficient *h* is evaluated by the model [15].

The first approach to get an idea about thermal treatment during grinding was an analytical approach by Carslaw and Jaeger [16]. They defined a two-dimensional heart source, moving with a constant velocity $v_{ft}$ and constant power over the workpiece surface. The heat generated by each grinding grain is summarized to one heat source. Since our workpiece is not homogenous because of the hole for the camera, an analytical solution will lead to inaccuracy.

Therefore we use a 3-dimensional numerical model for our calculations. In this model, the partial differential heat equation for solids

(2) $$\rho c_{p}\frac{\partial T}{\partial t}+\rho c_{p}u\nabla T+-\nabla \left(k\nabla T\right)=q$$

with the density ρ, the heat capacity $c_{p}$ the thermal conductivity *k*, the temperature *T* and the amount of energy *q* fed in the system or led off, is solved. Since grinding is a highly dynamic process, the model is calculated in a time-dependent study. The mesh was generated by tetrahedrons consisting of around 250.000 elements, with a higher resolution in the region of the sensor.

**Figure 5:**
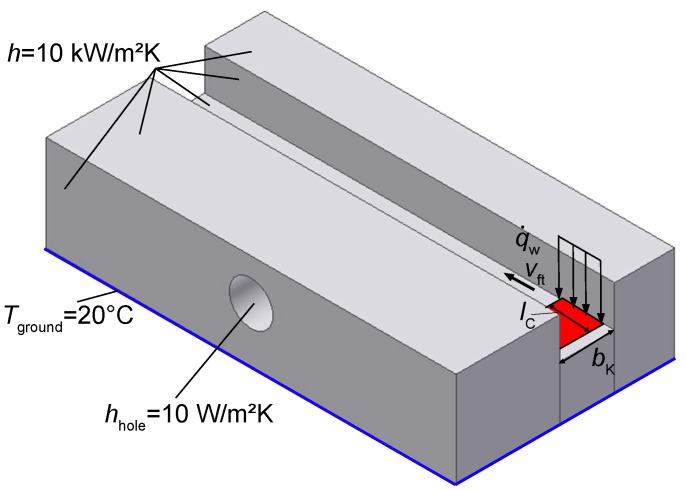
Boundary conditions used in the numercial model. All boundaries getting in contact with lubricant are set to convective heat transfer.

The model with its boundary conditions is shown in Figure 5. There are two main boundary conditions, which have significant influence on the results: the power of the moving heat source with the amount of energy dissipating in the steel and the thermal heat transfer coefficient *h* of the workpiece-lubricant interface.

Regarding the heat source, the heat flux density ${\dot{q}}_{w}$ can be calculated with the help of the tangential force $F_{t}$ measured during grinding (see Section 2) [15]:

(3) $${\dot{q}}_{w}=K_{v}K_{w}\frac{v_{c}F_{t}}{l_{g}b_{k}}$$

$l_{g}b_{k}$ is the area of the contact zone (contact length $l_{g}$ and the width of the grinding wheel $b_{k}$) and the cutting speed of the grinding wheel $v_{c}$. $K_{v}$ is a conversion factor, referring on how much of the total energy during grinding is converted into heat. In grinding terms, $K_{v}$ is approximately 1, because nearly the hole energy of the grinding wheel is converted into heat. $K_{w}$ is the heat distribution coefficient. This coefficient defines how much energy flows into the workpiece. $K_{w}$ is one of the fitting parameters which will be evaluated by our numerical model and the temperature measurement.

**Figure 6:**
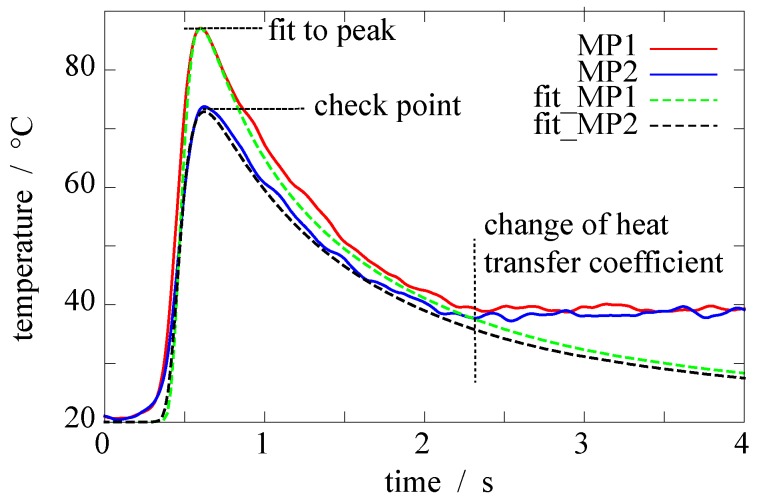
Measurement and fitting exemplary shown for measurement 7. The measurement results are given for the measurement points MP1 and MP2, according to the points given in Figure 3; the fitting results are shown with the dashed lines fit_MP1 and fit_MP2.

The heat transfer coefficient *h* defines the thickness of the boundary layer and thus the heat flux density out of the workpiece. Thus, a higher cooling rate leads to faster temperature decrease in the workpiece. A higher value of the heat transfer coefficient leads to higher cooling rate, which results in a steeper temperature decrease. This can be seen in Figure 6. After the temperature reaches a peak, the workpiece shows a characteristic curve of a cooling process. The thermal heat transfer coefficient *h* is also evaluated by the numerical model.

The procedure of evaluating $K_{w}$ and *h* can be explained by examining Figure 6. There, the locally measured temperature during grinding is plotted over time, in addition to the corresponding temperature in the model at the same point the measurement was done. To get to this, $K_{w}$ and *h* are adapted in the numerical model using a parameter study in a way that it fits the temperature curve. Therefore multiple comparable simulations are done. By changing the fitting parameters we can calibrate our simulation with the given measurement results. The more measurement we compare the merrier the simulation becomes. The peak temperature at the control point given in Figure 6 is used to double check the fit. This method is a common method to validate a simulation. The heat transfer coefficient *h* was varied from 2 $\frac{\mathrm{kW}}{m^{2}}$ to 40 $\frac{\mathrm{kW}}{m^{2}}$ in steps of 2 $\frac{\mathrm{kW}}{m^{2}}$. In case of h> 10 $\frac{\mathrm{kW}}{m^{2}}$, the resulting cooling curve in the simulation has a steeper slope than in the measurement. On the other hand, the cooling curve in the simulation decreases to slow in comparison to the measurement, when h< 10 $\frac{\mathrm{kW}}{m^{2}}$. As a result, h= 10 $\frac{\mathrm{kW}}{m^{2}}$ shows the best fit for all simulations, which is in good accordance to [17]. However, at t≈ 2.3 s the heat transfer conditions obviously change. There is a rapid cooling down from the peak temperature till t≈ 2.3 s with a constant *h*, showing good accordance with the fit by the model. Afterward, the heat transfer coefficient rapidly changes to very low values. This could be due to the structure of the grinding machine as seen in Figure 1. During grinding the lubricant nozzle is constantly flooding the workpiece with lubricant. After grinding, the nozzle has passed the workpiece and the lubricant supply is finally shut off; thus the workpiece is no longer supplied by fresh lubricant. In the numerical model, all boundaries which are in contact with lubricant are set to a constant convective heat transfer (*Neumann*) boundary condition, as seen in Figure 5. The bottom of the workpiece is set to a fixed temperature (*Dirichlet* boundary condition).

In our setup, the cutting speed of the grinding wheel is $v_{c}=$ 30 $\frac{m}{s}$. The contact length is calculated by the infeed and diameter of the grinding wheel: $l_{g}=\sqrt{a_{e}d_{gw}}$. For the three different infeeds $a_{e}=$ 20 μm, $a_{e}=$ 30 μm and $a_{e}=$ 40 μm the contact lengths are $l_{g20um}=$ 2.82 mm, $l_{g30um}=$ 3.45 mm and $l_{g40um}=$ 3.99 mm respectively. The parameters $F_{t}$ and ${\dot{q}}_{w}$ used in each simulation as well as the resulting heat distribution coefficient $K_{w}$ and the peak temperature in the contact zone are listed in Table 1. Regarding the heat source, we tested three different shapes: a source with constant heat over the entire contact length (according to Carslaw and Jaeger [16]), positive and negative triangular shaped heat source. We could not obtain any difference in the resulting temperature curve which might be due to low infeed of just a few microns and the high feed speed. Thus the shape of the heat source has negligible impact of the resulting heat distributions.

## 4. Experimental and Numerical Results

**Figure 7:**
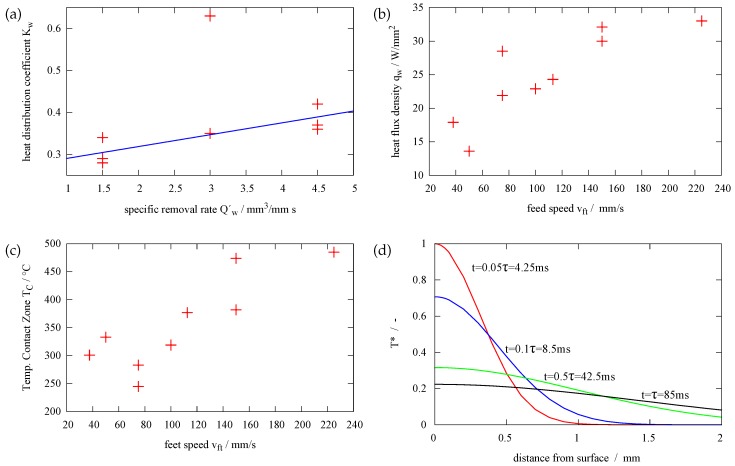
(**a**) The heat distribution coefficient $K_{w}$ depending on the specific removal rate $Q_{w}^{\prime }$. The blue fitting curve is given for all measurements without the outlier measurement no. 5 (**b**) heat flux density depending on the feed speed of the workpiece $v_{ft}$ (**c**) Temperature in the contact zone in dependency of the feed speed. (**d**) heat distribution in the steel at different times, the temperature $T^{*}$ is normalized to $T^{*}=\frac{T}{T_{max}\left(t=4.2\;\mathrm{ms}\right)}$.

The resulting heat distribution coefficients from Table 1 versus the specific removal rate are plotted in Figure 7 . In the diagram, the fifth measurement seems to be an outlier. There might be an incorrect setting during grinding, leading to a higher infeed, which could be possible in such low infeeds (this why the measurement is set into brackets in Table 1). Excluding measurement number 5 we can do a linear regression as shown by the solid line in Figure 7. By increasing the specific removal rate from $Q_{w}^{\prime }$ = 1.5 $\frac{{\mathrm{mm}}^{3}}{\mathrm{mm}s}$ to $Q_{w}^{\prime }$ = 4.5 $\frac{{\mathrm{mm}}^{3}}{\mathrm{mm}s}$ the heat distribution coefficient increases. This means that due to a higher specific removal rate, a higher percentage of the total energy during grinding increases. On the other hand, less energy is taken away by the chip, the lubricant and the wheel. The results are in good accordance with the predictions given in [15] by Brinksmeier et al.

If we now examine Figure 7b, the heat flux density ${\dot{q}}_{w}$ penetrating the workpiece increases with higher feed speeds. According to this, the peak temperature on the workpiece surface increases with increasing feed speed, as seen in Figure 7c. This contrasts with the temperature in the workpiece at MP1. If we look back to the measurement results in Figure 3c, increasing feed speed leads to lower temperatures in the workpiece.

In the first instance, we usually would assume higher temperatures in the workpiece, if the temperature in the contact zone increases. However, this is not the case. To understand the decreasing temperature, we need to examine the concept of the thermal relaxation time as explained by *Landau* and *Lifschitz* [18]. At the initial instant, a finite quantity of heat is concentrated in an infinitely thin layer. The heat then distributes in the material in a Gaussian shaped way, as seen in Figure 7d at different times *t*. The thermal relaxation time τ is then [18]:

(4) $$\tau =\frac{L^{2}}{\chi }$$

*L* is the characteristic length of the body and χ the thermal diffusivity ($\chi =\frac{\lambda }{\rho c}$). In our setup, we can define the characteristic length as the distance between the point of measurement MP1 to the grinding surface: L= 1 mm. The thermal diffusivity is χ= 11.7 × 10^−6^ $\frac{m^{2}}{s}$ (λ= 42.5 $\frac{WK}{m}$, ρ= 7720 $\frac{\mathrm{kg}}{m^{3}}$, c= 470 $\frac{Jk}{\mathrm{kg}}$). Finally, the thermal relaxation time for our system is τ= 85 ms. The temperature distribution as shown in Figure 7d can then be calculated by $T\left(x,t\right)=T_{max}\frac{1}{2\sqrt{\pi \chi t}}e^{\left(-x^{2}/4\chi t\right)}$ ($T^{*}$ is normalized to the peak temperature at t=4.2 ms and thus $T^{*}=\frac{T}{T_{max}\left(t=4.2\;\mathrm{ms}\right)}$) [18]. If we turn back to the heat source, the heat source during grinding at one specific point on the surface is switched on for $t_{impact}=\frac{l_{c}}{v_{ft}}$ (contact length divided by feed speed). An energy impact occurs. As seen in Table 1, the impact time for all grinding runs ($t_{impact,min}=$ 13 ms) is lower than the thermal relaxation time, except for grinding run 7 ($t_{7}=$ 103 ms). For higher grinding feed, we get shorter impact time. This means, when the heat reaches the point of measurement, the heat source above this point has already been switched off for several tens of milliseconds. Thus, for higher grinding speeds, the measurement point does not “see” the heating period due to the grinding wheel just above the sensor, however, the distribution in the steel after the grinding wheel has passed. In case of lower feed speeds, the heat source stays above the measurement point for a relatively long time, until everything is heated up. After the heat source has passed, cooling occurs.

Finally, for shallow grinding of thin layers in the range of $a_{e}=$ 20–40 μm we can state two things: First, increasing the feed speed will lead to higher heat flux densities in the workpiece and second, higher feed speed lead to higher temperature gradients between the grinding surface and points deeper in the material.

## 5. Conclusions and Outlook

We have presented a new measurement method to characterize shallow grinding processes. In the first part, we presented our measurement setup which consists mainly of an infrared camera integrated into a workpiece. Different grinding parameters were set up and temperature measurements conducted with the sensor. Initial measurement results indicated we found out that increasing the feed speed leads to lower temperatures in the workpiece. In the second part of the paper, we used a numerical model to investigate the grinding process in terms of the heat distribution in the workpiece. First, the heat distribution coefficient increases with the specific removal rate. Second, the heat flux density in the workpiece in the contact zone increases with increasing feed speed. Third, the temperature gradients increase with higher grinding feeds because the temperature in the contact zone increases while the temperature in the workpiece decreases.

In future work, we will do more experiments on grinding of such thin layers. Besides verifying our results, we will focus on the heat flux density as well as the thermal gradients in terms of damages in the subsurface zone. Microscopic images will help to inspect the grinding surface to see thermal damages in accordance material models and the behavior of steel due to thermal impact.

## Figures and Tables

**Table 1:** Parameters used in the numerical study and the resulting heat distribution coefficient $K_{w}$ the temperature in the contact zone $T_{C}$, the measured peak temperature $T_{\mathrm{peak}}$ at MP1 and the impact time $t_{\mathrm{impact}}$.

Measurement	$a_{e}$ /	$Q_{w}^{\prime }$ /	$v_{\mathrm{ft}}$ /	$F_{t}$ /	${\dot{q}}_{w}$ /	$K_{w}$ /	$T_{C}$ /	$T_{\mathrm{peak}}$ /	$t_{\mathrm{impact}}$ /
	μm	$\frac{{\mathrm{mm}}^{3}}{\mathrm{mms}}$	m/s	N	$\frac{W}{{\mathrm{mm}}^{2}}$	-	°C	°C	ms
1	20	1.5	75	116	64.6	0.34	301	86	38
2	20	3.0	150	154	85.8	0.35	333	54.6	19
3	20	4.5	225	160	89.1	0.37	283	47.4	13
4	30	1.5	50	107	48.7	0.28	245	75	69
5	30	3.0	100	80	36.4	0.63	319	49.7	35
6	30	4.5	150	196	89.1	0.36	377	51	23
7	40	1.5	37.5	157	61.9	0.29	382	40.7	106
8	40	3.0	75	207	81.5	0.35	474	46.9	53
9	40	4.5	112.5	147	57.9	0.42	485	34.5	35
